# Imaging and Metabolic Diagnostic Methods in the Stage Assessment of Rectal Cancer

**DOI:** 10.3390/cancers16142553

**Published:** 2024-07-16

**Authors:** Rafał Maksim, Angelika Buczyńska, Iwona Sidorkiewicz, Adam Jacek Krętowski, Ewa Sierko

**Affiliations:** 1 Department of Radiotherapy, Maria Skłodowska-Curie Białystok Oncology Center, 15-027 Bialystok, Poland; rmaksim@onkologia.bialystok.pl; 2 Clinical Research Centre, Medical University of Bialystok, 15-276 Bialystok, Poland; angelika.buczynska@umb.edu.pl (A.B.); adamkretowski@wp.pl (A.J.K.); 3 Clinical Research Support Centre, Medical University of Bialystok, 15-276 Bialystok, Poland; iwona.sidorkiewicz@umb.edu.pl; 4 Department of Endocrinology, Diabetology and Internal Medicine, Medical University of Bialystok, 15-276 Bialystok, Poland; 5 Department of Oncology, Medical University of Bialystok, 15-276 Bialystok, Poland; 6 Department of Radiotherapy I, Maria Sklodowska-Curie Bialystok Oncology Centre, 15-027 Bialystok, Poland

**Keywords:** rectal cancer, imaging diagnosis, stage assessment, CT, MRI, EUS, PET/CT, PET/MRI

## Abstract

**Simple Summary:**

Rectal cancer (RC) is a prevalent malignancy associated with significant morbidity and mortality. Accurate staging is crucial for optimal treatment and patient outcomes. This review examines current literature on imaging and metabolic diagnostic methods for RC staging. Key imaging techniques include magnetic resonance imaging (MRI), computed tomography (CT), and endorectal ultrasound (ERUS). MRI is the gold standard for local staging due to its superior soft tissue resolution and ability to assess tumor invasion depth, lymph node involvement, and extramural vascular invasion. CT is essential for detecting distant metastases and evaluating surgical resectability. ERUS provides detailed assessment of tumor depth, perirectal lymph nodes, and sphincter involvement. Understanding the strengths and limitations of each modality is vital for precise staging and treatment planning. Integrating multiple imaging and metabolic techniques, such as PET/CT or PET/MRI, enhances diagnostic accuracy and prognostic evaluation. This review assesses the effectiveness and accuracy of these diagnostic methods in RC staging.

**Abstract:**

Rectal cancer (RC) is a prevalent malignancy with significant morbidity and mortality rates. The accurate staging of RC is crucial for optimal treatment planning and patient outcomes. This review aims to summarize the current literature on imaging and metabolic diagnostic methods used in the stage assessment of RC. Various imaging modalities play a pivotal role in the initial evaluation and staging of RC. These include magnetic resonance imaging (MRI), computed tomography (CT), and endorectal ultrasound (ERUS). MRI has emerged as the gold standard for local staging due to its superior soft tissue resolution and ability to assess tumor invasion depth, lymph node involvement, and the presence of extramural vascular invasion. CT imaging provides valuable information about distant metastases and helps determine the feasibility of surgical resection. ERUS aids in assessing tumor depth, perirectal lymph nodes, and sphincter involvement. Understanding the strengths and limitations of each diagnostic modality is essential for accurate staging and treatment decisions in RC. Furthermore, the integration of multiple imaging and metabolic methods, such as PET/CT or PET/MRI, can enhance diagnostic accuracy and provide valuable prognostic information. Thus, a literature review was conducted to investigate and assess the effectiveness and accuracy of diagnostic methods, both imaging and metabolic, in the stage assessment of RC.

## 1. Introduction

Colon and rectal cancers (CRC) are the third most frequently diagnosed cancers in the United States and rank as the second leading cause of cancer-related deaths [1]. The incidence of rectal cancer (RC) is increasing globally, including in Poland, where it was ranked as the third most common malignancy diagnosed in 2023 [2,3]. The number of diagnosed cases has risen significantly since 1980, with a four-fold increase among men and a three-fold increase among women. In 2021, Poland reported 17,708 cases of CRC, with 40.1% of them localized in the rectum or rectosigmoid junction [3]. RC, like most CRCs, is predominantly sporadic, occurring in about 70% of cases with the average age of diagnosis after 50 years. Approximately 10% of cases demonstrate a hereditary pattern, posing a higher risk in patients under 50 years old, while another 20% exhibit familial clustering without a known inherited syndrome [4]. Risk factors for RC include a personal or family history of cancer, adenomatous polyps, and polyps with villous or tubulovillous dysplasia [5]. Patients with these factors are at increased risk for synchronous or metachronous primary CRC (up to 3% to 5% at five years) and require vigilant screening for RC [1]. Inflammatory bowel disease, particularly ulcerative colitis with rectal involvement, significantly increases the risk of RC. Studies indicate that the risk increases with the duration of the disease, reaching up to a 30% probability of CRC by the fourth decade after diagnosis. Additionally, a history of radiation therapy for prostate cancer has been associated with an increased risk of RC [6].

The clinical stage at the time of diagnosis is a crucial prognostic factor for RC. Patients diagnosed at stage I have a 5-year survival rate of 74.1%, while those diagnosed at stage IV have a 5-year survival rate as low as 6% [7,8]. Accurate assessment of the clinical stage is vital to determine the most appropriate treatment strategy based on the specific stage of the disease [9]. Surgical intervention is the preferred approach for early-stage disease, while neoadjuvant radiotherapy +/− sequential chemotherapy or radiochemotherapy followed by surgery is recommended for locoregionally advanced stages [10,11,12,13]. For oligometastatic patients, targeted treatments for metastases may include options such as surgical excision or stereotactic radiotherapy. Systemic treatment is offered for CS IV polimetastatic RC patients. Treatment decisions for metastatic disease are made on an individual basis within multidisciplinary teams [14,15,16]. Several diagnostic tools are currently available to determine the stage of RC, with some well-established in medical practice and others still evolving [9,17,18].

The primary objective of this review is to provide a comprehensive overview of the various imaging and metabolic diagnostic methods employed in the stage assessment of RC. By examining the existing literature, the study aims to evaluate the efficacy, accuracy, and limitations of these diagnostic tools in determining tumor stage, lymph node involvement, and the presence of distant metastases. The review encompasses a range of imaging modalities, which are discussed in detail. The review evaluates the strengths and limitations of each technique, their comparative effectiveness, and their role in guiding treatment decisions. By synthesizing the findings from multiple studies, this review aims to provide clinicians and researchers with a comprehensive understanding of the current landscape of imaging and metabolic diagnostic methods in the stage assessment of RC. The information presented in this review will help improve clinical decision-making, improve treatment planning, and ultimately contribute to better patient outcomes.

## 2. Materials and Methods

The PubMed database was utilized to perform a comprehensive literature review, adhering to the PRISMA and EQUATOR network guidelines [19,20,21,22]. Our analysis encompassed medical publications from 2000 to 2021. The systematic review titled “Imaging and Metabolic Diagnostic Methods in the Stage Assessment of Rectal Cancer” employed meticulously defined inclusion and exclusion criteria to ensure the selection of high-quality and relevant literature. The included studies were focused on imaging and metabolic diagnostic methods for RC staging, utilized robust research methodologies, provided comprehensive reporting of methods, results, and conclusions, and were published recently to incorporate the latest advancements in the field. Studies were excluded based on (1) findings not directly related to diagnostic and imaging methods in RC, (2) inadequate research methodologies, (3) incomplete reporting of findings, and (4) unclear and unreliable results. These criteria were rigorously applied to ensure the review included credible, current, and directly pertinent studies to enhance understanding of diagnostic and imaging approaches in RC staging. The selection and evaluation of articles were carried out independently, with the exclusion of studies that presented irrelevant conclusions, employed inappropriate research methodologies, exhibited inadequate reporting, or disseminated incomplete findings.

## 3. Transrectal Ultrasound (TRUS)

Transrectal ultrasound (TRUS), also known as endorectal ultrasound (EUS), involves the insertion of a specialized ultrasound probe into the rectum of the patient, which is covered with a gel-filled condom [23,24]. This technique is cost-effective and widely available, offering a detailed assessment of the anal sphincter structure and the depth of rectal wall infiltration within the scope of the examination [25].

In EUS images, five distinct layers can be identified. The first layer (hyperechoic) corresponds to the interface between the gel and the superficial layer of the mucous membrane. The second layer (hypoechoic) represents the lamina propria of the mucous membrane and the muscularis mucosae [23]. The third layer (hyperechoic) represents the submucosal layer and its interference patterns. The fourth layer (hypoechoic) represents the proper muscular layer. The fifth layer (hyperechoic) corresponds to the interface between the serosa and the mesorectal fat tissue [26].

EUS examination offers excellent visualization of the anatomical structure of the rectal wall, allowing for accurate assessment of the T stage according to the tumor (T), nodules (N), and metastases (M) (TNM) classification. It does not require the use of contrast agents and can be performed in patients with end-stage renal failure. A systematic review of 30 studies involving 4976 patients reported the accuracy of EUS in evaluating the T stage to range from 63% to 96%, with a mean value of 84%. In terms of the N stage, the accuracy of EUS ranged from 63% to 85%, with a mean value of 74%. However, a study conducted by Marusch et al. [27,28], which included 7096 RC patients from 300 German hospitals who underwent EUS prior to surgical intervention without neoadjuvant treatment, found the accuracy of local disease staging with EUS to be lower than previously reported in the literature.

The correlation between EUS assessment (uT) and histopathological examination (pT) of the T stage was demonstrated to be at an average level of 65%. This correlation varied depending on the annual EUS count in the centers: 63% in centers performing ≤10 EUS/year, 65% in hospitals performing 11–30 EUS/year, and up to 73% in hospitals performing over 30 EUS/year. The highest level of inconsistency between uT and pT comparisons was observed in cases of T2 and T4 stage RC, with 18% of cases underestimating and 17% overestimating the advanced stage of cancer in EUS [28]. In a similar prospective analysis conducted by the same team a few years earlier, the average diagnostic accuracy was 63% (51% for pT1 RC, 58% for pT2 lesions, 73% for pT3 tumors, and 44% for pT4 cancers) [29]. These findings indicate that EUS examination tends to overestimate the cancer stage in 24% of cases and underestimate it in 13% of patients. Both studies highlight a significant discrepancy between the data available in the literature and the actual results obtained in everyday clinical practice. EUS, as a diagnostic tool for assessing the stage of RC, is most effective in centers where the procedure is frequently performed as part of the diagnostic process. Recently, an interesting study comparing the sensitivity of EUS to magnetic resonance imaging (MRI) in evaluating extramural invasion (EMI) as a means of differentiating between T3a (EMI < 5 mm) and T3b (EMI > 5 mm) stages was published. The results of both imaging studies were compared with the results of histopathological examinations [28]. The diagnostic accuracy, sensitivity, and specificity of EUS for extramural invasion were reported to be 79.1–67.4%, 66.7–60%, and 85.7–82.1%, respectively. However, these results varied depending on the performing physician. In contrast, MRI examination showed better sensitivity, specificity, and diagnostic accuracy than EUS for differentiating T3a and T3b stages, with values of 80%, 89.3%, and 86%, respectively. Nonetheless, there were no significant differences in diagnostic accuracy between these examinations (*p* = 0.394 > 0.05; *p* = 0.124 > 0.05). The average diagnostic accuracy of EUS for overall T staging was reported to be 82–85%, while for N staging, it ranged from 70% to 75%. In comparison, MRI examination achieved an accuracy of 80.3% for T staging and 78% for N staging. These results significantly deviated from previous studies, likely due to the small sample size (*n* = 61) and the limited number of specialists evaluating the examinations, which may affect the interpretation [30].

Another study focused on evaluating lymph node involvement in the mesorectum in patients with T3-stage RC. A retrospective analysis of 289 patients who underwent neoadjuvant radiochemotherapy and 95 patients who did not receive this treatment found that EUS overestimated lymph node involvement in 6% of patients and underestimated it in 23%. This highlights the importance of not relying solely on EUS for selecting patients who require neoadjuvant radiochemotherapy to avoid unnecessary preoperative treatment [31,32].

In a retrospective study involving 219 patients with RC undergoing neoadjuvant treatment, differences were observed in the ultrasonographic assessment of uT and uN stages compared to the postoperative pathological assessment of tumor stage after neoadjuvant therapy (ypT) and lymph node status after neoadjuvant therapy (ypN) stages, depending on the distance of the tumor from the anal sphincter. Furthermore, EUS demonstrated significantly higher accuracy than computed tomography (CT) (64.8% vs. 34.7%, *p* < 0.001) [33,34,35,36]. The accuracy of EUS in evaluating the response to neoadjuvant treatment was found to be 100% for T0 in (20/20 cases), 43.8% for T1 (7/16 cases), 55.6% for T2 (30/57 cases), 78.0% for T3 (78/100 cases), and 65.6% for T4 (19/29 cases). Downstaging was observed in 53 cases (24.2%). The overall accuracy of determining the T stage using EUS was 70.3% (154/219 cases), which was significantly better than CT accuracy in this patient group. The accuracy of lymph node assessment in EUS for clinical N0 cases was 87.1% in 178 cases, 56.8% for N1 in 37 cases, and 100% for N2 (4 cases). Local downstaging was observed in only 4.6% (10 cases). The overall accuracy of assessing the N stage using EUS was 82.2% (180/219), which was significantly better than the clinical assessment of the N stage (82.2% vs. 60.7%, *p* = 0.006) [33,37,38]. The diagnostic accuracy varied depending on the location of the rectal tumor. Tumors located between 3–6 cm from the anal verge showed the most reliable assessment, with diagnostic accuracies of 78.0% for T, 88.6% for N, and 75.6% for the concordance between ultrasonographic and histopathologic staging. Despite these findings, there is evidence that the use of EUS without traditional CT imaging may lead to an increase in the number of patients undergoing neoadjuvant radiochemotherapy without a significant impact on overall survival [39,40].

In summary, EUS is a valuable method for assessing the stage of RC, especially in cases involving tumors in the distal segment of the organ. The application of EUS for precise staging evaluation is most accurate when the tumor is located 3–6 cm above the anal canal [41]. EUS examination, in addition to its accessibility and low cost, has several limitations. It cannot be used as the sole imaging modality for RC and serves as a useful adjunct in the diagnostic and therapeutic process [42,43].

## 4. Computed Tomography (CT) in the Diagnosis of Metastatic Tumors

**Figure 1:**
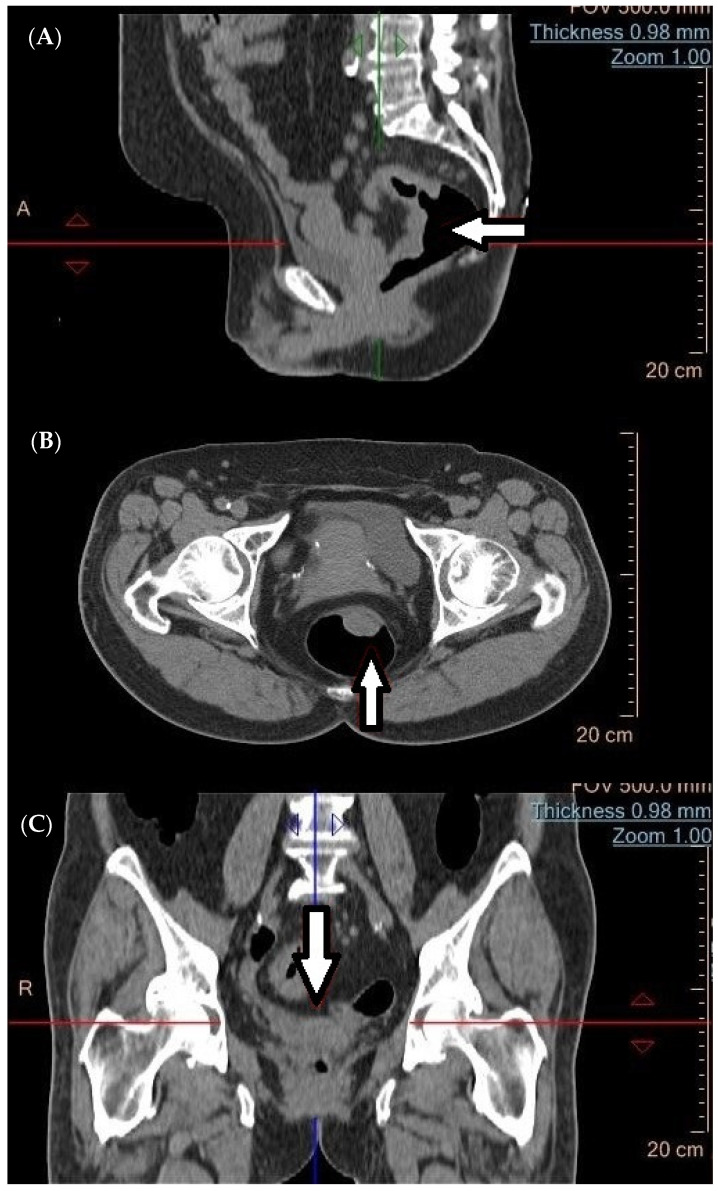
A rectal tumor (arrow) detected using CT in the (**A**) sagittal, (**B**) transverse, and (**C**) coronal planes, respectively.

In the previous chapter, the advantages and limitations of EUS in assessing local extent (T) and lymph node involvement (N) in tumors were discussed. However, CT has been introduced in clinical diagnostics as a promising tool, considering its role in identifying metastatic spread (M). CT, like EUS, finds widespread use in tumor diagnosis, but its application varies depending on the diagnostic goal [41]. Concerning the determination of T and N, CT may be suboptimal compared to more advanced techniques such as EUS or MRI. This is attributed to CT’s lesser capability in visualizing internal tissue layers, especially in soft tissues (Figure 1) [42]. However, CT excels in detecting distant metastasis. With its ability to perform rapid and precise whole-body scanning, CT can effectively identify tumor metastases in various organs. This is particularly significant in advanced tumors where there is a risk of cancer cells spreading beyond the primary site [44]. With its capacity for whole-body scanning and precise organ visualization, CT can detect metastases in various locations [45]. In the case of RC, metastases often manifest in the liver and lungs. CT proves notably useful in detecting RC-associated liver metastases [46]. The liver is well visualized on CT scans, facilitating the identification of metastatic lesions, even if they are small. Studies demonstrate CT’s high effectiveness in detecting liver metastases, enabling prompt and effective therapeutic interventions [47]. 

Lung metastases can also be identified using CT–CT images that allow precise assessment of lung structure and identification of tumor-related changes. However, the sensitivity for detecting lung metastases might be slightly lower than for liver metastases due to respiratory motion and the limited ability to differentiate certain changes [48]. When comparing CT’s ability to detect metastases in different organs, some distinctions become apparent [49]. In the case of CRC or lung cancer, CT exhibits higher sensitivity for detecting metastases in the liver compared to the lungs. This discrepancy arises from anatomical factors and the liver’s rich blood supply, facilitating early detection of metastases [50]. In clinical practice, it is an indispensable tool, especially in advanced tumors where rapid detection and treatment of metastases are crucial for patient prognosis. In the context of this examination, significant emphasis is placed on the diagnostic importance of early and late contrast phase imaging. Early contrast phase CT, typically obtained within 30–40 s after contrast injection, emerges as a crucial tool for identifying arterial-phase enhancement patterns within metastatic liver lesions. Notably, this phase proves invaluable in detecting small lesions and characterizing their vascularity [51]. On the other hand, late contrast phase CT acquired several minutes after contrast administration plays a vital role in assessing the washout characteristics of liver metastases from RC. This phase is instrumental in differentiating hypervascularity from hypo vascular lesions and monitoring the response of these lesions to therapeutic interventions [52]. The combination of early arterial-phase and late-phase CT imaging significantly enhances diagnostic precision in the field of liver metastases from RC. While arterial-phase images highlight the enhancement of these lesions, late-phase images reveal crucial information about contrast washout dynamics, thus facilitating the differentiation between malignant and benign lesions [51]. Radiologists rely on a dual-phase CT approach, incorporating both early and late contrast phases, to enable meticulous diagnosis, treatment planning, and vigilant monitoring of disease progression associated with liver metastases from RC [53]. In clinical practice, it is an indispensable tool, especially in advanced tumors where rapid detection and treatment of metastases are crucial for patient prognosis.

## 5. Virtual Colonoscopy (CTC)

**Figure 2:**
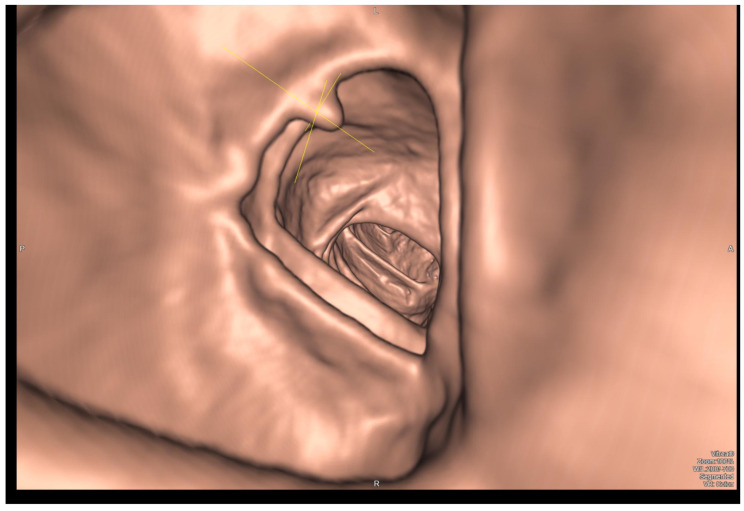
Virtual Colonoscopy (CTC). The image depicts a polyp in the large intestine (arrow).

Virtual colonoscopy (CTC, computed tomography colonography) is a procedure similar to conventional CT, with the difference that the patient is prepared for the examination as in a traditional colonoscopy, meaning that the colon needs to be emptied [54]. Additionally, during the procedure, a special rectal catheter is used to introduce carbon dioxide or air into the colon to inflate it [55]. In practice, carbon dioxide is more commonly used. Air, despite being less expensive, is not absorbed by the rectal mucosa and is less tolerated by the patient. Image acquisition is performed in both supine and prone positions, allowing for optimal gravitational compression of the intestinal wall and facilitating differentiation between polyps and residual fecal matter [56]. The obtained images can be reconstructed into a three-dimensional model of the colon, ready for evaluation by a radiologist (Figure 2). Another advantage of CTC is that it requires lower levels of radiation exposure compared to traditional X-ray examinations when imaging the large intestine only [57]. However, if there is a need to assess surrounding organs, appropriate protocols can be used to expand the examination to include a conventional abdominal CT scan without the need for a separate procedure. In recent years, the role of CTC as a potential alternative to endoscopic examination of the large intestine has been extensively studied. This procedure is used in patients with already diagnosed RC to assess the clinical stage and as a primary diagnostic tool for individuals who cannot undergo or have not completed an optical colonoscopy (OC) for various reasons. Studies are being conducted to achieve what is known as “digital bowel cleansing” [58]. By marking the stool with a special iodine marker and utilizing the different radiological properties of air, fecal masses, and the rectal wall, along with appropriate software for smoothing and reconstructing images from CT scans, it is possible to digitally “remove” the stool from the images and assess the intestinal wall. Such a tool would be extremely useful for patients with sensory impairments or those who have difficulties in tolerating large volumes of oral bowel preparation solutions required for proper preparation for a conventional OC [54].

A meta-analysis conducted by Plumb et al. [59], based on 622 patients, showed that in individuals for whom OC is not feasible but who have positive results for occult blood in stool, performing a CTC is justified. The average sensitivity for detecting adenomas larger than 6 mm, RC, and CRC using CTC is 88%, while the average specificity of this examination is only 75.4% [59]. Interesting results were the utility of OC and CTC as screening tests for detecting CRC in asymptomatic patients were performed [59]. The combined group of 3881 patients were analyzed for the diagnostic accuracy of OC and CTC, as well as the ratio of invitations to the actual number of examinations performed. A total of 8104 patients were invited for OC and 7310 patients for CTC. Among this group, 2333 individuals opted for CTC (28.8%), while 1486 (20.3%) chose to undergo OC. In individuals with positive CTC results, OC was performed as the gold standard for diagnosing CRC. In total, only 243 patients underwent both procedures. High-grade dysplasia was confirmed in 134 individuals, allowing for a positive predictive value of CTC ranging from 30.7% to 73.1%, depending on the performed study [60,61]. CTC is a valuable examination for the initial diagnosis of suspected colon lesions, primarily due to its good patient tolerance. Its use should be considered in cases where OC is contraindicated. However, it should be emphasized that OC remains the gold standard in CRC diagnosis.

## 6. Magnetic Resonance Imaging (MRI)

Numerous reports of relatively low specificity, sensitivity, and diagnostic accuracy of CT, particularly in determining the T stage and evaluating the response to neoadjuvant treatment, have led to the clinical preference for MRI over CT in patients with RC [62]. This examination allows for the identification of several unfavorable factors that can be more accurately assessed compared to CT, such as the depth of tumor infiltration beyond the intestinal wall, the margin of unaffected tissue around the tumor, the extent and location of adjacent organ involvement, and the presence of extramural venous invasion (EMVI), manifested by the presence of tumor signal in the vascular system beyond the muscularis propria [10,63]. In a study conducted on 63 patients who underwent an MRI of the pelvic area before planned surgery, the results of this radiological examination were compared with the histopathological findings, revealing the high sensitivity (89.3–100%) and specificity (76.9–100%) of MRI. The observed wide ranges of values are due to the varying accuracy of MRI in assessing different anatomical regions of the small pelvis [64,65]. Groundbreaking data were obtained from the prospective MERCURY study, which included as many as 295 patients. The difference between EMVI assessment in MRI and histopathological examination was only 0.5 mm, which is significantly sufficient for planning optimal surgical intervention [66]. After a 5-year follow-up of the aforementioned patient group, it was found that the MRI determination of the circumferential resection margin (CRM) status could be a better prognostic factor for assessing the risk of local recurrence (LR) than the TNM staging [67]. In a group of 374 patients, LR occurred in 36 (9.6%), which was significantly lower than previously reported. Subgroup analysis revealed that patients with threatened CRM in MRI had a 20% risk of LR, while in the group with confirmed involved CRM in histopathological examination, LR occurred in 27% of patients. In contrast, LR was observed in only 7% of cases in patients with unthreatened CRM in MRI or histopathological examination. Patients with involved CRM in both MRI and histopathological examination had the worst prognosis, with disease-free survival (DFS) and LR rates of 25% and 32%, respectively. The prospective analysis of the MERCURY II study aimed to assess whether MRI can accurately determine the clinical stage of the disease and the likelihood of achieving a clear CRM in histopathological examination. This could lead to a reduction in the risk of non-radical surgery and, on the other hand, a decrease in the number of permanent stomas performed [65]. Among 326 patients diagnosed with RC located within 60 mm above the anal sphincter, the clinical stage was assessed by performing and analyzing MRI. Based on the results, patients were divided into a “safe” group and a “threatened” group regarding the involvement of the surgical margin. Additionally, radiologists assessed several features that could have prognostic significance, such as EMVI, depth of invasion, lymph node status, the distance of the tumor from the anal sphincter, and determination of the involved quadrant of the rectum (defined as the wall with the greatest tumor invasion on axial MRI) [10,68]. Assignment to a specific group determined the selection of a particular treatment scheme (Table 1).

In the studied patient group, among those who did not require preoperative treatment and had a safe CRM on MRI assessment (n = 88), a negative histopathological margin was achieved in 86 cases (97.73%) postoperatively. Among patients who received neoadjuvant treatment (n = 78), a negative margin was obtained in 72 patients (92.31%) [69]. In the group of patients with a threatened CRM on MRI assessment who underwent neoadjuvant treatment, a follow-up MRI was performed, and three subgroups were identified. The first group of patients showed a good response to neoadjuvant treatment (n = 33): 100% of cases had clear histopathological margins; the second group of patients showed a poor response to neoadjuvant treatment (n = 46): 76.09% of cases had clear histopathological margins; the third group of patients went without a clinical stage assessment after neoadjuvant treatment (n = 13): 76.92% of cases had clear histopathological margins [69,70].

Among individuals with a threatened CRM on MRI assessment who did not undergo preoperative treatment (n = 21), involvement of the histopathological margin was found in 14.29% of cases. All results were statistically significant. Subgroup analysis revealed additional factors influencing the risk of margin involvement that can be determined using MRI: tumors located within 4cm of the anal sphincter carry a 3.4-fold risk of margin involvement, but in the absence of other unfavourable factors, the risk of postoperative margin involvement is 4%. EMVI increases the risk of margin involvement by 3.8 times. Tumors located in the anterior quadrant of the rectum carry a 2.8-fold risk of margin involvement [52,71]. The study identified the primary limitation of MRI as the steep learning curve and the requirement for multidisciplinary teams to create a personalized treatment plan. In the absence of proper protocols, standard MRI reports frequently lack crucial prognostic information necessary for making optimal treatment decisions [72].

In a meta-analysis focused on the diagnostic efficacy of MRI in assessing regional lymph node invasion, a comprehensive systematic review was conducted, encompassing studies published between 2000 and 2021. These studies encompassed diverse MRI protocols, countries of origin, and various reference standards for assessing lymph node metastasis. The results of the meta-analysis revealed that the sensitivity and specificity of MRI in diagnosing lymph node metastasis were 0.73 and 0.74, respectively, with an AUC value of 0.7877. Subgroup analysis indicated that both high-frequency (3.0 Tesla) and high-resolution MRI demonstrated higher sensitivity and specificity compared to low-frequency (1.5 Tesla) MRI. Additionally, a significant impact of blinded methodology during study quality assessment was identified [73].

MRI exhibits high sensitivity and specificity in detecting liver metastases. Studies have consistently demonstrated MRI’s superiority over other imaging modalities in terms of sensitivity, with reported values as high as 93.1% in detecting secondary hepatic lesions. Moreover, MRI offers improved specificity compared to CT, with values reaching 87.3%. This heightened sensitivity, and specificity make MRI, particularly when enhanced with hepatobiliary contrast agents, such as gadolinium-ethoxybenzyl-diethylenetriamine pentaacetic acid (Gd-EOB-DTPA), a valuable tool for accurately diagnosing liver metastases. Additionally, MRI’s ability to provide detailed anatomical and functional information further enhances its utility in lesion detection and characterization, contributing to improved patient outcomes and treatment planning [74].

## 7. Metabolic Imaging Using Positron Emission Tomography/Computed Tomography (PET/CT)

PET/CT has emerged as a valuable and comprehensive imaging modality in the evaluation of RC, offering insights into both metabolic activity and anatomical details for precise diagnosis and staging. Furthermore, the versatility of PET/CT allows for the utilization of various tracers, enabling a range of imaging approaches for a deeper understanding of tumor biology and treatment response.

### 7.1. 2-Deoxy-2-[fluorine-18]fluoro-D-glucose Tracer (18F-FDG)

For years, new diagnostic possibilities have been sought in PET/CT, most commonly with 18F-FDG, for both primary staging assessment and evaluating response to treatment or resolving ambiguous imaging results in the assessment of LR. Even though CT imaging in PET/CT is based on low-dosage cone beam CT, it provides valid clinical information [75]. Data on the use of 18FDG-ET/CT in assessing RC are scarce, and most analyses are based on patient groups with a diagnosis of CRC without isolating those with rectal tumors [76]. A published analysis in 2011 of 30 studies on the utility of 18FDG-PET/CT in the diagnosis of CRC did not find sufficient evidence (two studies were excluded based on small patient groups) to recommend routine use of this examination in the preoperative assessment of RC [77]. For the evaluation of LR, five studies were analyzed, showing an average sensitivity and specificity of 91%. For assessing distant metastases, the average sensitivity and specificity were 91% and 76%, respectively, emphasizing the low quality of the analyzed studies [77]. In 2014, a retrospective study based on 67 RC patients who underwent 18FDG-PET/CT before treatment planning was conducted [78]. Among the entire group, the examination results led to a change in the treatment approach for 20 patients compared to the planned treatment based on standard diagnostic tests. In three cases, radical treatment was abandoned in favor of palliative care due to revealed metastases that were invisible or less numerous in the CT scan [78]. In three cases, the treatment plan was changed from palliative to radical due to the lack of confirmation of liver metastases initially identified in the CT alone scan but not confirmed in the PET scan. In the remaining 14 patients, the change in the treatment plan concerned the scope of therapy but not the change from palliative to radical therapy or vice versa. Interestingly, synchronous neoplastic disease in another location was detected in four patients, two of whom had lung cancer, while the other two had adenocarcinoma of the pituitary gland and diffuse large B-cell lymphoma (DLBCL). Among the remaining patients (n = 40), 18FDG-PET/CT results corresponded to other imaging findings in 34 cases, while in six patients, additional or smaller changes were detected compared to conventional imaging, but the results did not affect the planned treatment strategy. Due to the retrospective nature of the study, no consensus was reached regarding the routine use of 18FDG-PET/CT in the preoperative diagnosis of CRC [78]. In 2017, a study focusing on 18FDG-PET/CT in the assessment of metastatic lymph nodes in patients with CRC was published. A total of 370 patients who underwent preoperative 18F-FDG PET/CT were included in the analysis [79]. The study demonstrated unsatisfactory sensitivity of the 18FDG-PET/CT at 56.8% but good specificity at 90.3% and diagnostic accuracy of 74.2%. For contrast-enhanced CT, the respective values were 38.4%, 95.5%, and 65.0%. This confirms the ability of 18FDG-PET/CT to confirm tumor involvement in lymph nodes that were ambiguously identified in other imaging studies. An interesting study from the current year presents new possibilities for 18FDG-PET/CT image reconstruction using point spread function reconstruction (PSF). Fifty-nine patients with RC who underwent 18FDG-PET/CT, PSF-PET/CT, and contrast-enhanced MRI before surgery were analyzed. The obtained results were compared with histopathological findings. The PSF-PET/CT showed a significantly higher sensitivity for determining the N feature (78.6%) compared to 18FDG-PET/CT and MRI, which had sensitivities of 64.3% and 57.1%, respectively. All diagnostic methods exhibited high specificity, ranging from 93.5% to 96.7%. There were no significant differences between the examinations in determining the T feature, while both conventional 18FDG-PET/CT and PSF-PET/CT provided accurate assessment of distant metastases in all recorded cases [80]. The continuous evolution of medical imaging techniques has unveiled a promising avenue in the realm of diagnosing and managing CRC. Notably, PET scans, a sophisticated imaging modality, have emerged as a pivotal tool in this context [81]. Traditionally, FDG, a radiotracer that mirrors glucose metabolism, has been the primary choice for PET imaging. However, the dynamic landscape of medical research has ushered in a new era, expanding the horizons of PET imaging by introducing diverse tracers with specific biological affinities [82]. This evolution addresses the limitations posed by FDG, as certain types of tumors might exhibit variable glucose metabolism rates. Here are a few examples of other tracers utilized in PET imaging for the diagnosis of CRC.

### 7.2. 18F-FLT (Fluorothymidine) PET/CT

This tracer measures the levels of DNA replication within cells. Increased DNA replication is present in cancer cells; thus, 18F-FLT PET/CT can assist in identifying regions of elevated tumor activity. The study was performed by McKinley et al. [83] and investigated the potential of 18F-FLT PET imaging to predict early treatment response in patients with KRAS wild-type RC undergoing neoadjuvant therapy. The study used 18F-FLT PET/CT to measure DNA synthesis, aiming to identify if changes in tumor proliferation can predict treatment outcomes. Following the combined treatment of cetuximab and chemoradiotherapy, all patients exhibited a significant reduction in 18F-FLT PET uptake, exceeding 70% when compared to their baseline measurements. This reduction in 18F-FLT uptake demonstrated a strong correlation with the achievement of a complete pathological response in the patients. Consequently, the study posits that 18F-FLT PET emerges as a highly promising imaging biomarker for anticipating the response to neoadjuvant therapy, particularly when cetuximab-mediated EGFR blockade is incorporated in individuals diagnosed with RC. This pilot study aimed to establish the feasibility of 18F-FLT PET/CT as a tool for early response assessment, potentially leading to more tailored treatment strategies for RC patients.

### 7.3. 68Ga-DOTATATE (Gallium-68 DOTATATE) and 68Ga-DOTANOC PET-CT

While more commonly used for neuroendocrine tumors, 68Ga-DOTATATE may have applications in imaging rectal neuroendocrine tumors, which are relatively rare. It targets somatostatin receptors expressed in certain types of tumors [84]. The study investigates the effectiveness of 68Ga-DOTATATE PET/CT in diagnosing recurrent neuroendocrine tumors. In 31 patients (44%), the presence of recurrent neuroendocrine tumors (NETs) was confirmed through histopathology or follow-up examinations. Recurrent NET metastases were mainly found in the liver (n = 14), lymph nodes (n = 8), lung (n = 4), bones (n = 3), soft tissue (n = 3), or as local recurrences (n = 14). Additionally, three patients had tumors of non-neuroendocrine origin (one each of non-Hodgkin lymphoma, signet-ring carcinoma, and CRC). 68Ga-DOTATATE PET/CT successfully detected NET recurrence in 28 out of 31 patients and ruled out recurrent NET in 33 out of 39 patients, resulting in a sensitivity of 90% and specificity of 85%. Another study investigated the utility of two imaging techniques, 68Ga-DOTANOC PET-CT and 18F-FDG PET-CT, in detecting primary rectal neuroendocrine tumors and regional lymph node metastases. 68Ga-DOTANOC PET-CT showed a sensitivity of 89.58% in detecting primary RNETs. 18F-FDG PET-CT had a sensitivity of 77.08% for primary tumor detection. The combination of both techniques increased sensitivity to 93.75%. 68Ga-DOTANOC PET-CT demonstrated a sensitivity of 77.78% and a specificity of 91.67% in detecting LN metastases. 18F-FDG PET-CT had lower sensitivity (38.89%) but higher specificity (100%) for LN metastasis. The study suggests that 68Ga-DOTANOC PET-CT is more reliable than 18F-FDG PET-CT for detecting primary rectal NETs, especially in G1-G2 rectal NETs. However, both techniques have limitations in detecting LN metastases [53].

### 7.4. Future Perspectives of PET/CT

These innovative tracers, customized for specific molecular processes and pathways implicated in CRC, have the potential to transform cancer imaging. By pinpointing specific biomarkers or molecular characteristics linked to malignancies, these tracers offer a refined perspective on tumor biology. Regarding 64Cu-ATSM (copper amino acid complex), this tracer demonstrates the capability to accumulate within regions of hypoxic (oxygen-deprived) tissue, frequently found within tumor masses. This attribute could assist in pinpointing areas of tumors experiencing insufficient oxygen supply [85]. 18F-DOPA (Fluorodihydroxyphenylalanine), while commonly used in neuroendocrine tumor studies, has also been evaluated in CRC research. This tracer is associated with amino acid activity and might assist in detecting tumor areas [86].

## 8. Metabolic Imaging 18F-FDG PET/MRI

**Figure 3:**
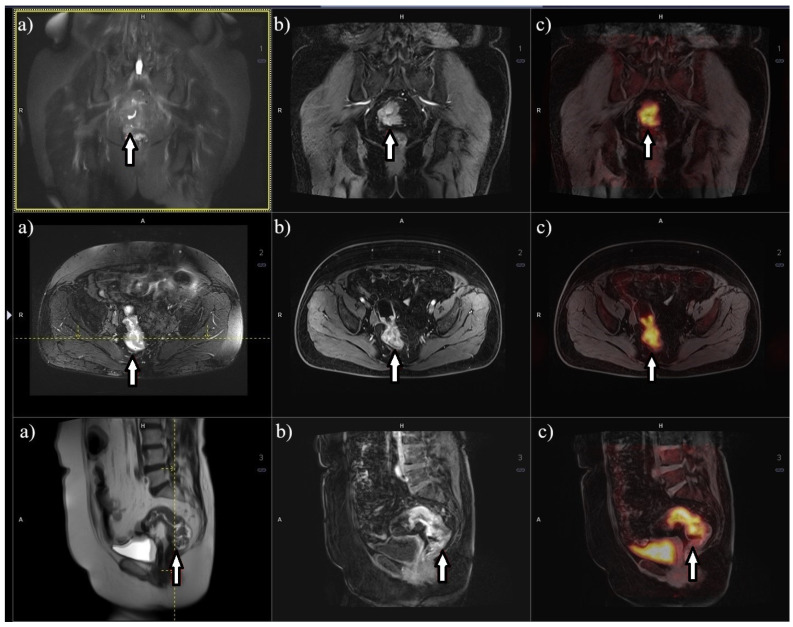
PET/MR images on a 3-tesla Biograph mMR Siemens^®^ scanner using fluorodeoxyglucose labeled with fluorine (18F-FDG). The images show rectal cancer (arrow), respectively: (**a**) MR in T2-weighted sequence, (**b**) MR in T1-weighted sequence, and (**c**) fusion of MR images in T1-weighted sequence and PET.

PET/MRI is an innovative imaging technique that combines MRI and PET, most commonly with an 18F-FDG tracer. The clinical advantages of PET/MRI primarily arise from the supplementary information provided by MRI compared to CT and the fusion of PET and MRI images. When compared to PET/CT, PET/MRI offers several significant benefits [87]. Firstly, MRI in 18F-FDG PET/MRI serves as a comprehensive diagnostic examination, whereas CT in 18F-FDG PET/CT is merely low-dose cone-beam computed tomography used for anatomical localization of areas with heightened radioisotope metabolism. Additionally, 18F-FDG PET/MRI does not employ ionizing X-ray radiation and enables diffusion-weighted imaging and higher tissue resolution, facilitating more precise characterization of the examined tissues (Figure 3) [88]. However, 18F-FDG PET/MRI does possess some drawbacks in contrast to 18F-FDG PET/CT, including a 3–5 times higher cost, considerably longer imaging duration, and contraindications for MRI due to the presence of metallic implants, endoprostheses, stimulators, and similar factors within the patient’s body [89].

In 2015, a study comparing the diagnostic accuracy of PET/MRI and PET/CT in determining the staging of RC demonstrated a significantly higher precision of PET/MRI in determining the T feature [90]. Concerning the N feature, there is a possibility that a dedicated MRI scan in PET/MRI can provide moderately accurate results for RC. PET imaging, in this case, can furnish supplementary information regarding glucose metabolism, thereby increasing the likelihood of detecting affected lymph nodes. Furthermore, PET/MRI can facilitate the assessment of treatment response in neoadjuvant therapy. Evaluating tumor regression based on MRI can assist in determining survival time. Moreover, a decrease in the standardized uptake value (SUV) in the tumor after chemoradiotherapy corresponds to an increased probability of a complete treatment response [91].

In a study conducted by Kang et al. [92] in 2016, 51 patients with RC underwent PET/MRI and multidetector CT, and the results were compared. PET/MRI yielded additional information in 14 out of 51 patients (27.5%) in comparison to CT, encompassing improved visualization of extracolonic lesions in 12 out of 51 (23.5%) cases and the identification of additional findings outside the colon in 2 out of 51 (3.9%) cases. Consequently, the treatment strategy was altered for 11 out of 51 (21.6%) patients [93].

## 9. Discussion

The evaluation of imaging studies in RC can be influenced by several key factors described in this review. Firstly, the importance of implementing standardized protocols and training programs could impact reducing variability in diagnostic accuracy [94]. The quality and consistency of interpreting results can be significantly enhanced when specialists are adequately trained and adhere to procedural standards in diagnostics [95]. Another factor is the cost–benefit analysis of various diagnostic methods. Economic aspects can significantly influence decisions regarding the selection of specific diagnostic techniques. The costs and effectiveness of these imaging studies in both clinical and economical contexts are crucial for assessing their utility and implementation in clinical practice [96]. These aspects need to be considered as they may constitute significant limitations during comparable imaging studies [97]. Conducting a detailed cost assessment is challenging due to the diversity of healthcare systems globally and the evolving regulations governing medical service financing and reimbursement [98]. Furthermore, longitudinal studies are needed to allow for the assessment of the long-term impact of various diagnostic methods on patient health outcomes. Such studies can provide data on the long-term therapeutic effects and patient survival, which are crucial for evaluating the effectiveness of imaging diagnostics [99]. Finally, the role of a multidisciplinary team in the diagnostic process of RC should be emphasized. The integration of specialists from various fields, such as radiologists, oncologists, and surgeons, can significantly improve comprehensive patient management and optimize therapeutic outcomes [100]. All these factors must be considered when evaluating and implementing imaging studies in the diagnosis of RC to ensure the best possible care for patients. Therefore, this review defines all diagnostic methods for assessing T stage and N stage accuracy in RC, serving as a comprehensive resource that summarizes the strengths of various approaches based on current literature. Table 2 outlines all specific diagnostic imaging methods employed, such as MRI, CT scan, PET-CT, and EUS, and evaluates their accuracy in determining both the T stage and the N stage (Table 2).

## 10. Improving the Future of Imaging Diagnostic Procedure Performance

The integration of artificial intelligence (AI) technologies has emerged as a promising avenue in the field of RC diagnostics [107]. AI algorithms are capable of analyzing complex imaging data with exceptional accuracy and efficiency, thereby facilitating early detection of lesions, precise characterization of tumors, and effective treatment planning strategies [108]. By harnessing machine learning and deep learning techniques, AI can sift through vast amounts of radiological and pathological data, identifying subtle patterns and anomalies that may not be readily apparent to human observers [109]. The application of AI in radiology not only enhances diagnostic precision but also holds the potential to streamline workflows and improve clinical decision-making processes [107]. Automated image analysis and interpretation by AI systems can expedite the reporting process, reduce diagnostic errors, and optimize resource allocation in healthcare settings [110]. Furthermore, AI-powered predictive models can assist clinicians in predicting patient outcomes and personalized treatment strategies based on comprehensive data analysis [111]. In the context of RC, the per-patient receiver operating characteristic curve (ROC) for radiomics models was 0.808 (0.739–0.876), while for deep learning models, it was 0.917 (0.882–0.952), both significantly higher than the radiologists’ ROC of 0.688 (0.603–0.772). Similarly, for colorectal cancer, radiomics models achieved a per-patient ROC of 0.727 (0.633–0.821), outperforming the radiologists’ ROC of 0.676 (0.627–0.725). These findings suggest that AI models have the potential to more accurately predict lymph node metastasis in both rectal and colorectal cancer. However, it is important to note that radiomics studies are heterogeneous, and there is a scarcity of deep learning studies, indicating a need for further research to standardize methodologies and validate findings [112]. Nevertheless, in conjunction with multidisciplinary team collaboration, where radiologists, oncologists, and surgeons cooperate synergistically, AI technologies complement and augment human expertise, leading to more informed decisions and enhanced patient care [110,113]. As AI continues to evolve and integrate into clinical practice, ongoing research and validation studies are essential to ensure the reliability, safety, and ethical considerations of these technologies in enhancing the diagnostic capabilities and overall management of RC patients [108,114]. Introducing international protocols with deep learning AI technology holds promise in mitigating these challenges and, importantly, reducing human error. AI-powered standardized approaches can enhance diagnostic consistency and reliability, transcending geographical and professional disparities. By automating image analysis and interpretation, AI systems have the potential to improve diagnostic accuracy and streamline workflows, thereby optimizing patient care [115]. Addressing these limitations through collaborative efforts and standardized AI-driven protocols represents a pivotal step towards enhancing diagnostic precision and clinical outcomes in global healthcare practices [116].

## 11. Limitation of the Study

Limitations of the study include the challenge of selecting the optimal diagnostic tool due to the inherent strengths and weaknesses of each method. Several of the referenced studies also have small sample sizes, which could impact the reliability of the received results. Importantly, each diagnostic modality captures different variables and aspects of RC, making direct comparisons complex [117]. Moreover, disparities in technology across countries result in varying imaging capabilities and quality, impacting diagnostic accuracy [117]. Additionally, healthcare professionals’ diverse educational backgrounds and different levels of procedural and interpretative experience contribute to variability in diagnostic outcomes [118]. Furthermore, financial considerations play a significant role, with reimbursement policies varying widely between healthcare systems globally [119].

## 12. Conclusions

Imaging techniques, including EUS, CTC, CT, MRI, PET/CT, and PET/MRI, play a crucial role in the diagnosis and monitoring of treatment effectiveness in RC patients. EUS, CTC, and MRI provide detailed anatomical images, allowing for the evaluation of tumor spread and invasion into adjacent structures. CT offers anatomical imaging and aids in disease staging. PET is particularly useful for assessing glucose metabolism and identifying areas of increased tumor activity. In recent years, the integration of PET with other modalities, such as CT (PET/CT) or MRI (PET/MRI), has significantly improved the diagnosis and assessment of RC. PET/CT combines metabolic data with accurate anatomical images obtained from CT scans. PET/MRI combines metabolic information with high-resolution anatomical images, enabling precise localization of metabolically active regions. These imaging techniques facilitate tumor staging, detection of lymph node metastases, evaluation of neoadjuvant therapy effectiveness, and monitoring of treatment response. Each technique has its own advantages and limitations, and the choice depends on the specific case and available resources. To summarize, EUS, CTC, MRI, and PET are highly valuable in the diagnosis and monitoring of RC. Their utilization enables a comprehensive evaluation of disease progression and assists in making appropriate treatment decisions. The integration of different modalities, such as PET/MRI and PET/CT, further enhances diagnostic capabilities and provides a more comprehensive clinical perspective. Furthermore, by underscoring persistent human error, financial and equipment limitations, and training constraints, the advancement of AI in medical imaging shows considerable potential to enhance the accuracy of cancer diagnosis and prognosis.

## Figures and Tables

**Table 1:** Recommended management for early-stage rectal cancer, according to the assessment of local cancer advancement and mesorectal fascia (MF) involvement on magnetic resonance imaging (MRI) [47].

Interstitial Space Status in MRI	MF Status in MRI	Distance of Invasion from the Levator Ani Muscle	Recommended Surgical Treatment
Safe: tumor above the inter-sphincteric space	Tumor > 1 mm from MF	>10 mm	Inter-sphincteric resection ± anastomosis
Safe: tumor limited to the submucosal layer, with the preserved full thickness of the muscularis propria	Tumor > 1 mm from MF	≤10 mm	Local excision
Safe: tumor involving a portion of the muscularis propria	Tumor > 1 mm from MF	≤10 mm	Inter-sphincteric resection ± anastomosis
Endangered: any of the following is sufficient: involvement of the entire thickness of the muscularis propria/internal anal sphincter involvement of the inter-sphincteric space invasion of the internal anal sphincter or <1 mm from the levator ani muscle	Tumor > 1 mm from MF	≤10 mm	Abdominopelvic excision of the rectum—extralevatory abdominoperineal excision (ELAPE)
Safe or Endangered	Infiltration beyond the MF into adjacent organs: prostate bladder vagina sacrum pelvic fascia		Prolapse of the small pelvis

**Table 2:** Diagnostic accuracy of imaging and metabolic methods in T and N staging of rectal cancer.

Diagnostic Method	T Stage Accuracy	N Stage Accuracy	Strengths	Limitations	Literature
EUS	63–96%	63–85%	Cost-effective and widely available Detailed anatomical assessment No contrast agents required High accuracy for T staging Effective for specific tumor locations	Variable accuracy in N staging Inter-operator variability Limited accuracy for high rectal tumors Tendency to overestimate or underestimate stages	[17,26,36]
CT	38.7–86%	60.70%	Effective for detecting distant metastases Rapid and precise whole-body scanning	Suboptimal for T and N staging Limited anatomical resolution	[50,57,101]
MR	76–100%	73–78%	High sensitivity and specificity Accurate assessment of unfavorable factors Better prognostic value Enhanced diagnostic accuracy Reduced risk of non-radical surgery Effective in liver metastasis detection	Steep learning curve Long acquisition time Protocol dependency Challenges in lymph node assessment Technical limitations	[94,102,103]
PET/CT	N/A	74.20%	Comprehensive imaging modality Versatility with various tracers Metabolic activity insight Evaluation of lymph nodes and metastases Detection of synchronous neoplastic diseases Potential for early treatment Response prediction	Limited specific evidence for rectal cancer Variable sensitivity and specificity Mixed results in treatment Planning impact Technical limitations Tracers’ variable efficacy Requirement for a multidisciplinary approach	[82,103,104]
PET/MRI	92–100%	42–92%	Comprehensive diagnostic examination High tissue resolution Diffusion-weighted imaging No ionizing radiation Improved visualization of extracolonic lesions Impact on treatment strategy Metabolic activity insight Evaluation of lymph nodes and metastases	Cost Longer imaging acquisition Contraindications Learning curve and interpretation Limited availability	[103,105,106]

EUS—endorectal ultrasound; CT—computed tomography; MR—magnetic resonance imaging; PET/CT—positron emission tomography/computed tomography; PET/MRI—positron emission tomography/magnetic resonance imaging.

## Data Availability

Not applicable.
